# Digitally supported HIV self-testing increases facility-based HIV testing capacity in Ekurhuleni, South Africa

**DOI:** 10.4102/sajhivmed.v23i1.1352

**Published:** 2022-06-13

**Authors:** Nolundi T. Mshweshwe-Pakela, Tonderai Mabuto, Luke Shankland, Alex Fischer, Dikeledi Tsukudu, Christopher J. Hoffmann

**Affiliations:** 1 Department of Implementation Research, The Aurum Institute, Johannesburg, South Africa; 2 School of Public Health, University of the Witwatersrand Johannesburg, South Africa; 3 Aviro Health, Cape Town, South Africa; 4 Department of Health Systems, The Aurum Institute, Johannesburg, South Africa; 5 Department of Medicine, Johns Hopkins University School of Medicine, Baltimore, United States of America; 6 Department of Health, Behavior, and Society, Johns Hopkins Bloomberg School of Public Health, Baltimore, United States of America

**Keywords:** HIV testing, HIV self-testing, facility-based HIV testing, digital support, linkage to ART

## Abstract

**Background:**

HIV testing is the first step for linkage to HIV prevention or treatment services. Facility-based HIV testing is the most utilised method, but faces challenges such as limited work space and human resources. Digitally supported HIV self-testing (HIVST) provided in clinics shifts testing to the client, potentially empowering the client, and addresses such constraints.

**Objectives:**

The study primary objective was to determine the feasibility of integrating digitally supported HIVST into the clinic. Secondary objectives were to describe HIV testing volume, populations reached, and antiretroviral treatment (ART) initiation.

**Method:**

We conducted an analysis of prospectively collected data during implementation of digitally supported HIVST in two healthcare facilities based in South Africa from June 2019 to September 2019. We described implementation and client characteristics using HIVST and compared testing before and during implementation.

**Results:**

During the 4-month implementation period there were 35 248 client visits. A total of 6997 (19.9%) of these visits involved HIV testing. Of those testing, 2278 (32.5%) used HIVST. Of the 2267 analysed, 264 (11.6%) were positive: 182 (12%) women and 82 (11%) men. Of those, 230 (95.4%) were confirmed HIV positive and 150 (65%) initiated ART within 14 days. During a four-month pre-implementation period, 14.5% of the clients tested for HIV. Compared to the pre-implementation period, we observed a 25% increase in HIV testing.

**Conclusion:**

Digitally supported HIVST increased the number of clients completing HIV testing in the health facility, without a need to significantly increase staff or space. Facility-based digitally assisted HIVST has the potential to increase HIV testing in high HIV prevalence clinic populations.

## Background

HIV testing is the first step in linkage to HIV care, including prevention or treatment services.^1,2^ The United Nations Joint Programme on HIV/AIDS has set interim 95-95-95 targets: that 95% of people living with HIV know their HIV status, 95% of these are to be initiated onto antiretroviral treatment (ART), and that 95% of these should be virally suppressed by the end of 2030.^3^ By the end of 2019, HIV testing services (HTS) had reached about 87% of people living with HIV (PLHIV)in the Eastern and Southern African region and up to 92% of the overall population in South Africa.^4^ Of all HTS strategies, facility-based HTS is the mostly widely utilised, with 70% of all testing in South Africa occurring in public clinics and hospitals.^5^ Individuals who test HIV positive during facility-based testing have higher subsequent engagement in care, presumably because they are already seeking clinical care and interacting with the medical system.^6,7^ However, challenges such as limited space and limited human resources are major constraints to the daily test volume.^8,9^ A second testing challenge is limited acceptability of standard HTS for some clinic clients.^10,11,12^ Innovative strategies could increase testing volume and improve outreach to a greater proportion of clinic clients.

In 2015, the World Health Organization recommended the addition of HIV self-test (HIVST) as a complementary approach to standard HTS.^13,14^ South Africa updated its HIV counselling and testing guidelines and adopted this recommendation.^15^ HIV self-test is a process whereby a lay-person collects his or her own specimen (usually a buccal mucosa swab), performs the test, and interprets the result.^16^ It is generally acceptable and is preferred by some clients, especially from hard-to-reach populations.^17^

HIV self-test shifts testing to the client, potentially empowering the client, while also reducing human resource and space demands on the clinic. If conducted within a clinic, HIVST has the potential to substantially increase the overall HIV testing capacity of the facility. Considerations for HIVST centre around a client’s ability to accurately collect the specimen, conduct the test, and interpret HIV results.^18,19^

HIV self-test using OraQuick test kits has shown high acceptability due to its non-invasive and easy-to-perform nature; however, some clients express the need for assistance, which may not be possible when the test is conducted without a health worker present during testing.^20,21^ In addition, post-test guidance may support the client’s health journey, and confirmatory testing is needed for positive HIVST results as part of the HIV testing algorithm.

In prior work, we identified that < 10% of patients visiting the facility received HIV testing.^9^ A major barrier to increasing testing was lack of space and testing personnel. In response to these challenges, we developed a digitally supported HIVST system for facility-based use. The digital platform was designed to shift testing guidance from personnel and to provide standardised guidance and counselling content, thus overcoming the limited supply of HIV counsellors. Self-testing by clients in small kiosks maintained privacy while overcoming the challenge of limited space from counsellor-led testing. The digital support is a software application installed on a digital tablet that provides content on conducting HIVST, steps after testing, and living with HIV, as well as a countdown timer for the testing process. This software was co-created by Aviro and The Aurum Institute to define and assess a delivery model of digitally supported HIVST in a health-facility setting. The primary objective of this study was to determine the feasibility of integrating digitally supported HIVST into the clinic; secondary objectives were to describe HIV testing volume, populations reached, and ART initiation.

## Methods

We collected data from February 2019 to May 2019 prior to implementing HIVST (baseline), and from June 2019 to September 2019 during pilot implementation of digitally supported HIVST (implementation), for a before-and-after comparison of HIV testing volume and HIVST use.

### Setting

The study was conducted in the Ekurhuleni district located in the Gauteng province of South Africa, outside the city of Johannesburg. In 2019, the City of Ekurhuleni had a population of 3 774 638 and a land size of 1975 km². Ekurhuleni comprises urban and peri-urban residential areas with a total of 93 public health clinics and 6 public hospitals. All public health facilities provide HTS free of charge. This study was conducted in two public community healthcare facilities. Both facilities are in urban areas and each had an average daily headcount of 400 clients; each operated for 8 hours per day. Both facilities had primary care providers who were professional nurses, with a medical doctor visiting up to three days per week.

These facilities provide primary health services, including acute and chronic care, family planning, antenatal care and childhood immunisations. In both facilities, free HTS were provided by trained lay counsellors.

One facility had five, and the other six, lay counsellors. Both facilities allocated two rooms/workspaces for HTS inside the facility and two foldable gazebos outside the facility that provided additional private space for HTS delivery. These facilities were selected in coordination with the district-level Department of Health to pilot the digitally supported HIVST.

### HIV testing services

Posters regarding HIV testing were prominently displayed in the clinics and health talks were conducted in waiting areas during baseline and implementation periods. During the implementation period these talks informed clients about standard testing provided by counsellors and HIVST using OraQuick.

### Standard HIV testing

When a client accepted standard HIV testing, they were sent to a counselling room or a gazebo where a counsellor would provide the service to one client at a time. Standard HTS takes a median of 26 min, as we have described from prior work in public clinics in this district.^9^

### HIV self-testing

**FIGURE 1:**
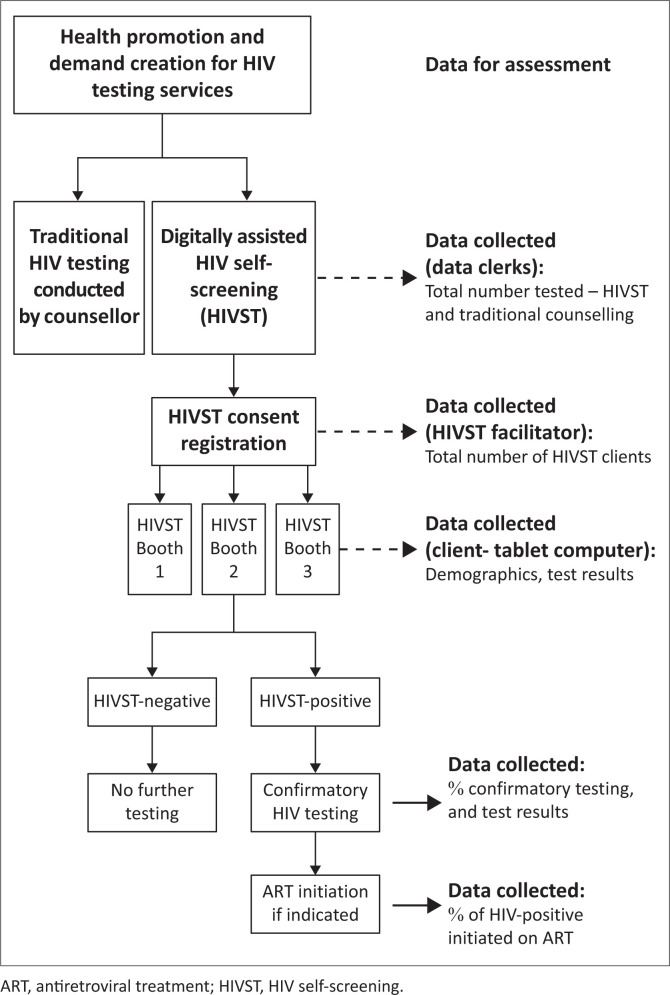
Facility HIV testing flow incorporating digitally supported HIV self-test.

OraQuick information brochures, translated into several local languages, were distributed to clients in waiting areas to give them more information about self-testing. Figure 1 shows the client flow from information to completion of confirmatory testing.

Three 1 m × 1 m HIVST booths were placed inside each health facility. Each booth had pictorial instructions to guide the testing process, an OraQuick^®^ HIV Rapid Antibody Test (OraSure Technologies, Inc., Pennsylvania, United States [US]), a tablet device with the digital application, and headphones for clients to listen to audio content on the digital application. OraQuick is a lateral flow test for antibodies using a specimen from an oral mucosal swab.

**FIGURE 2:**
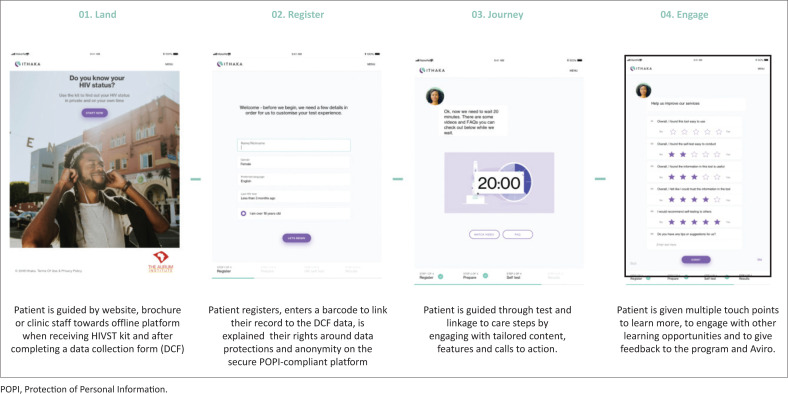
Digital support journey.

Each facility had a dedicated HIVST facilitator, trained in HIV testing and the digital platform and allocated to facilitate digitally supported HIVST. HIV self-test facilitators requested written consent for HIV testing, and a separate written consent to use the digital application, as it was implemented as a pilot study. HIV self-test facilitators also assisted clients when called upon. HIV self-testing was supported by a digital application delivered through a tablet device in each HIVST booth (Aviro Pocket Clinic, Aviro Health, Cape Town, South Africa) that provided audio-visual content via the screen and attached headphones. The digital support sought to guide a client’s HIVST journey from testing to next steps after a negative or positive test. The journey started with the client agreeing to terms of use and data collection and registering demographic information (Figure 2).

Clients were then guided through pre-test counselling, testing (including a video demonstrating the use of the OraQuick HIV test kit), and post-test counselling. The content features, voice, and examples were tailored to the client’s age and sex as entered at the start of the session. After self-sample collection from the buccal mucosa, the client was prompted to set the on-screen timer. During the 20 min waiting period for the OraQuick results, the client was led through audio-visual health-related content regarding HIV and HIV testing. After the 20-min OraQuick development time was completed, the client was asked to enter the test result. Further audio-visual post-test counselling was provided based on the entered test result. The client also had the option to get assistance from the HIVST facilitators.

Individuals who tested positive were reminded of the need for a confirmatory test as per the South African algorithm. If the client agreed, the facilitator escorted the client to a lay counsellor to conduct a confirmatory test. If they declined testing, the facilitator documented this. If both screening and confirmatory test results were positive, linkage to HIV care was initiated.

### Data collection

Data were retrospectively abstracted from clinic paper testing registers to identify unique individuals, testing outcome (positive or negative), sex and age grouping.

HIV self-test facilitators, funded by the study, recorded sex, age, history of HIV testing, and HIV test results for all clients opting for HIVST. For those patients with a positive HIV test result with HIVST, we abstracted available ART data from the electronic record system and patient paper files (we did not assess linkage to ART for patients receiving routine HTS). Facility headcounts were tabulated daily by clinic staff using a register that listed all clinic clients.

Abstracted data were queried for missing information. Clinic source documents were reviewed to resolve queries and update the study database.

### Analysis

We sought to describe operational and technical feasibility based on whether the HIVST kiosks could be used within the overall clinic flow and whether their use was sustained during the pilot period (e.g. without equipment breakdown), whether clinic clients would use the HIVST system, whether the digital tablet assistance was used by clients, and whether testing was completed with results. This was based primarily on the experience of the HIVST facilitators and supported by results of HIVST volume. Data were analysed using STATA^©^ version 16 (2019, StataCorp LLC, College Station, Texas, US). We calculated the median age of the patients testing for and diagnosed with HIV before and during the implementation phase. Additionally, numbers and proportions were used to report categorical variables. We further calculated percentages of HIV confirmatory test outcomes and linkage to ART for the categories listed above. We also calculated percentages for HIV test volume and test outcome for the baseline and implementation periods. We used the chi-square test to compare the proportion of clients receiving HTS pre-implementation and while HIVST was being implemented.

### Ethical considerations

An application for full ethical approval was made to the Witwatersrand Human Research Ethics Committee and ethics consent was received on 11 February 2021. The ethics approval number is 201111. All procedures performed in this study were in accordance with the ethical standards of the institutional research committee and with the 1964 Helsinki Declaration and its later amendments. Additionally, as per HIV testing guidelines, all clinic attendees who participated in the Aviro Pocket Clinic HIVST provided consent to HIV testing and counselling. For this analysis, all data variables were de-identified.

## Results

During the 4-month pre-implementation baseline period, there were 34 393 client visits, with a total of 4999 (14.5%) involving HTS. The majority of clients testing for HIV were women (***n*** = 3474; 69.5%); 541 (10.8%) tested HIV positive. The median age of those testing positive was 34 years (interquartile range [IQR]: 28–40 years).

During the 4-month implementation period, there were 35 248 client visits in the two health facilities. A total of 6997 (19.9%) of these visits involved HIV testing. Of those testing, 2278 (32.6%) used HIVST. We excluded 11 patients from the analysis because they were below 18 years of age. Among the 2267 clients who used HIVST, 1535 (67.7%) were women (Table 1). The median age was 28 (IQR: 24–33 years). The median age of those testing positive was 33 years (IQR: 27–38 years). The majority of clients using HIVST were aged 18–35 years (Table 1).

**TABLE 1:** Characteristics of patients who used the Aviro Pocket Clinic HIV self-test platform in the two pilot health facilities.

Variable	*n*	%
**Gender**		
Female	1535	67.70
Male	732	32.30
**Age**		
≤ 24	675	29.80
25–35	1090	48.10
36–49	412	18.20
≥ 50	82	3.60
Missing	8	0.35
**HIV status**		
Reactive	264	11.60
Non-reactive	2003	88.40

*N* = 2267.

### HIV diagnosis

Of the 2267 clients that used HIVST, 1535 were women and 732 were men (Table 1). A total of 264 (11.6%) were positive on OraQuick, with a similar proportion of women (182/1535; 12%) and men (82/732; 11%) testing positive. One hundred and thirty-five/264 (51.1%) of those testing HIV positive were aged 25–35 years old; 18/264 (6.1%) were aged ≥ 50 years.

Of the 264 clients who screened HIV-positive, 241/264 (91.3%), received a documented HIV confirmatory test, of which 230/241 (95.4%) were confirmed to be HIV-positive. Of those who were confirmed HIV positive, 150/230 (65%) initiated ART at the same clinic within 14 days; overall, 184/230 (80%) initiated ART within nine months at the same clinic at which they had the testing.

### Role of digitally supported HIV self-test in overall facility HIV testing services

**FIGURE 3:**
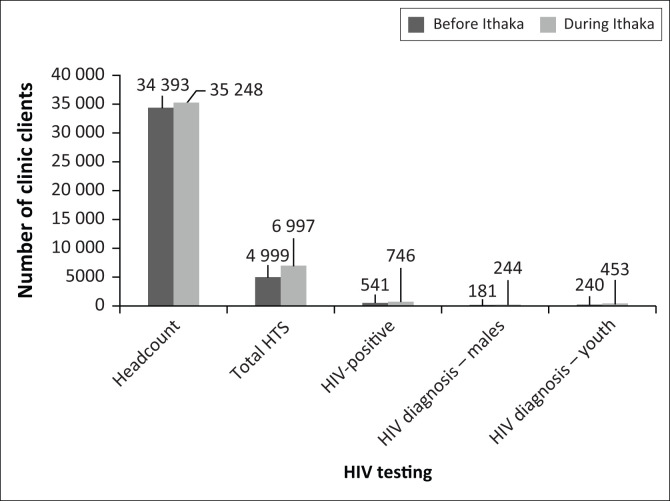
Comparison of facility HIV testing services before and during Ithaka (1 February 2019 – 30 September 2019).

The HIVST programme increased overall facility HIV tests of patients aged 15 years and above by 25% (14.5% vs 19.9% of clients testing; chi-square *P* < 0.001), while maintaining a stable HIV testing yield of 11% (Figure 3). The HIVST positivity yield was 12% – similar to traditional HTS. Importantly, the use of this platform almost doubled the number of youth (aged 18–35) diagnosed with HIV, increasing from 240 to 453 (Figure 3).

## Discussion

Clinic-based digitally supported HIVST increased HIV test volume without decreasing the HIV testing yield in a pilot study in two clinics in South Africa. Clinic-based HIVST was able to increase volume without requiring increased space and with only a modest increase in human resources.

HIV self-test has considerable promise that includes demonstrated acceptability^22,23^ and access to more difficult-to-reach populations.^24,25^ Our study builds on prior work of digitally supported HIVST that took place in patients’ homes. Using this method, it was found that 70% patients logged into the digital application, and 22% reported their HIV test results. The acceptability of digital HIVST support has previously been reported from a qualitative study.^26^ A meta-analysis of digital support for HIV testing noted that digital support increased HIVST when compared with its absence.^27^ The acceptability and usability of the specific digital support platform used in this study (Aviro Pocket Clinic, previously named Ithaka^28^), as well as other digital platforms used in community or clinic testing, have previously been reported.^26,27,28^ Prior studies of facility HIVST have reported an increase in HIV testing compared to standard HIV testing. A study of facility-based HIVST that did not use digital assistance required a considerable increase in healthcare worker resources and increased space to complete the testing.^29,30^

Compared to HIVST conducted away from health services, health facility-based HIVST has the value of being able to rapidly provide confirmatory HIV testing and initiate ART among those testing positive.

Notably, studies of HIVST conducted away from health services often have limited data on HIV test outcome, confirmatory testing, and linkage to ART.^22,31^ Facility-based HIVST has the additional value of being able to directly engage clients in prevention programmes, such as pre-exposure prophylaxis for those at high risk.

In South Africa and similar settings, space and personnel limitations are a major constraint on the capacity of routine HIV testing.^9,32,33^ With digitally supported clinic-based HIVST, the client manages testing and content can be tailored to the client’s age and gender. This shift from healthcare worker to client testing considerably reduced the human resource requirement, enabling one health worker to provide self-testing support to multiple individuals in private kiosks situated inside a clinic. It is plausible that empowering the client with testing may enhance subsequent care engagement. The digital support element allowed for a uniform content that promoted HIV prevention and the HIV care continuum engagement. We believe that this has the potential to overcome counselling quality and content challenges with current lay-counselor-provided post-test counselling.^34^

The strength of this study is the integration and assessment of HIVST into routine care offered by regular service delivery personnel in public clinics, with minimal intrusion by study staff.

A limitation is that only two sites were studied for a relatively short period of 4 months and we relied on a comparison of pre-test data at the same sites over a similarly short period. In addition, we relied on routinely collected data (collected through routine health systems and the Aviro Pocket Clinic). This limited the range of available data and may have affected data quality. For example, we only had age data from the pre-implementation period for individuals who tested HIV positive and not all individuals accessing HTS. Finally, pre and post comparisons are subject to secular trends unrelated to the study intervention. We conducted the study over a short timeframe which limits the potential of this confounder, but does not eliminate it.

## Conclusion

Use of technology to support the HIV care continuum from HIVST to ART initiation to retention in care has the potential to contribute to improved proportions reached along the HIV care continuum. Reaching updated 95-95-95 targets will require the use of novel and innovative care engagement approaches. Our data support the proposition that digitally supported HIVST platforms (and, potentially, other components of the care continuum) enable the shift of the care dynamic toward the client and client-centred care. Further studies of HIVST implementation and scale-up and digital support platform optimisation are needed to increase HIV testing and diagnosis to reach current targets.
